# Abortion knowledge and experiences among young women and men in Accra, Ghana

**DOI:** 10.12688/gatesopenres.12961.2

**Published:** 2020-09-07

**Authors:** Sarah T. Reiger, Phyllis Dako-Gyeke, Thoai D. Ngo, Gillian Eva, Leonard Gobah, Kelly Blanchard, Sruthi Chandrasekaran, Kate Grindlay

**Affiliations:** 1 Ibis Reproductive Health, Cambridge, MA, 02140, USA; 2 Social and Behavioural Sciences, School of Public Health, College of Health Sciences, University of Ghana, Accra, Ghana; 3 Poverty, Gender and Youth Program, Population Council, New York, New York, 10017, USA; 4 Marie Stopes International US, Washington, DC, 20033, USA; 5 Marie Stopes Ghana, Accra, Ghana

**Keywords:** Medication abortion, abortion access, sexual and reproductive health, family planning, urban youth, Accra, Ghana

## Abstract

**Background** **:** Despite the presence of legal abortion services in Ghana, unsafe abortion remains common, particularly among young women. Little is understood about what young people know about safe and legal abortion, and if and how they are utilizing it.

**Methods** **:** To characterize abortion use and address gaps in safe access, from September-December 2013, we conducted a cross-sectional survey with 100 men and 250 women aged 18-24 in Accra, Ghana. Participants were asked about abortion experiences, including prior services, providers, methods, satisfaction, perceived support, and knowledge of laws. Descriptive statistics, Fisher’s exact tests, and chi-square tests were performed.

**Results** **: **Among surveyed youth, most (87% of women, 64% of men) thought abortion was illegal or did not know the law. In total, 30% of women and 14% of men ever had an abortion and partner who had an abortion, respectively. Among women’s most recent abortions, medication abortion (61%), surgical methods (26%), and unsafe methods categorized “least safe” (14%) were the initial or only methods used. Most women who accessed medication abortion initially or as their only method saw a pharmacist (40%) or no one (33%). Nearly one-quarter of women (n=16, 24%) who initially took tablets used more than one method.

**Conclusions** **:** Despite experiences with abortion, most young people in this study were unaware of its legality and unsafe abortions occurred. More needs to be done to ensure young people understand the law and have access to safe methods, and that pharmacists are trained to provide appropriate doses and formulations of medication abortion.

## Introduction

Globally, one in ten pregnancies ends in unsafe abortion ^1^. In Ghana, despite liberalization of abortion law in 1985, which states that abortion is legal when performed by a registered medical practitioner in cases of rape, incest, risk to the life or health (mental or physical) of the woman, or fetal abnormality ^2^, unsafe abortion is still common ^3^. In fact, unsafe abortion, defined as ‘a procedure for terminating an unintended pregnancy carried out either by persons lacking the necessary skills or in an environment that does not conform to minimum medical standards, or both ^4^, is the second single leading cause of maternal death in Ghana ^5^. The country’s most recent maternal mortality ratio was estimated to be 310 deaths per 100,000 livebirths ^6^, and it is thought that 11–30% of those deaths are associated with unsafe abortion ^7^. Young women in Ghana are especially at risk, as adolescents are 77% less likely to have a safe abortion compared to women aged 30 and older ^8^.

Recognizing the impact of unsafe abortion on Ghana’s high rates of maternal mortality, the Government of Ghana launched the Reducing Maternal Mortality and Morbidity (R3M) program in partnership with a consortium of international health organizations in 2006 to improve access to family planning and comprehensive abortion care services ^9^. As part of this effort, more was done to disseminate information about the abortion law, train providers, and introduce modern technologies for abortion care. This included getting temporary approval by the Ghana Food and Drug Board for mifepristone ^10^, a medication abortifacient that can be used in conjunction with misoprostol ^11^, an oxytocic drug already Food and Drug Board-approved.

Medication abortion drugs, mifepristone and misoprostol, are increasingly available globally, and their combination is now not only included on the World Health Organization model list of essential medicines ^12^, but also the Ghana Essential Medicines List ^13^. However, medication abortion may involve self-administration of drugs at home, and this potential use outside of the formal medical system adds a layer of complexity to the concept of abortion “safety.” ^14^ There is anecdotal evidence that women in Ghana purchase mifepristone and/or misoprostol directly from pharmacies and use these drugs for medication abortion. Provision of medication abortion drugs via pharmacies may improve the accessibility and acceptability of abortion in resource-poor and restrictive settings. However, evidence for its utilization, effectiveness, safety, and acceptability among young people in Ghana is lacking. Questions also remain about what young people know about safe and legal abortion, and if and how they are utilizing it. This study assessed abortion knowledge and experiences among young Ghanaians living in Accra to identify gaps in access to safe and satisfactory reproductive health services.

## Methods

### Study design and sample

From September to December 2013, four trained fieldworkers working in pairs administered cross-sectional surveys randomly, available as *Extended data* ^15^, to eligible participants of the same sex and approximate age. Survey interviews were conducted in private locations at the recruitment sites, which included market places, social clubs, and sports venues in Accra, Ghana (Kokomlemle and Tema New Town). Eligible participants were aged 18–24 years, spoke English, Twi, or Ga, and were sexually active in the six months prior to the survey. The survey collected information about socio-demographic and reproductive health characteristics, contraceptive knowledge, use, and perceptions, and experience with unintended pregnancy and abortion. Questions differed slightly between male and female questionnaires to account for personal versus sexual partner experiences. Field workers obtained informed consent from each participant prior to initiation of the survey, which took approximately 45–60 minutes to complete. Participants were reimbursed 10 Ghanaian Cedi (GH¢) (approximately $3.00 USD). The study instruments were piloted prior to initiation of data collection, and periodic quality control checks were done throughout data collection. The study received institutional review board approval from the Nouguchi Memorial Institute for Medical Research at the University of Ghana, Legon (114/12-13) and the Marie Stopes International Ethical Review Committee (011-13). Sampling methodology and findings on contraception use and unintended pregnancy are reported elsewhere ^15^. Data was not collected on how many people were overall approached versus participated.

### Data analysis

For this study, our primary outcomes of interest were the percentage of women who reported prior abortion and the proportion of women using each specific abortion method. Other variables reported in this study included demographic and sexual and reproductive health characteristics, knowledge of abortion law, and details about abortion utilizers’ most recent experience: reason for abortion, method, provider, location, support, satisfaction, post-abortion contraception, and adverse events. Information was also collected from young men regarding any sexual partners’ abortion experiences.

Participants’ self-reported abortion methods used were categorized into medication abortion, which included responses of misoprostol or “tablets”; surgical abortion, which included unspecified surgical methods and dilation and curettage (D&C) (no respondent specifically selected vacuum aspiration); or known unsafe methods ^15^. While we recognize that abortion safety is a spectrum, contingent not only upon the utilized method, but also the skills of the provider, medical standards of the facility, and use of proper evidence-based protocols and medications, we were unable to assess such information. Thus, in this study, we assessed use of a known unsafe abortion method at any time during participants’ most recent and coded the following methods that were used as unsafe methods: injection, ingestion of specific liquids or herbal concoctions, insertion of herbs or other objects into the vagina and heavy massage.

Descriptive statistics and chi-square tests were calculated. Fisher's exact test was used when any cell count was less than or equal to five. A level of p<0.05 was considered as statistically significant. Data were stratified by sex where appropriate; bivariate analysis were conducted by initial/only abortion method. All statistical analyses were conducted using Stata 14 (StataCorp, College Station, Texas). 

## Results

### Study population

In total, 250 women and 100 men participated in the survey. Underlying data are available from Harvard Dataverse ^15^. Demographic and reproductive health characteristics of the participating women and men are shown in Table 1 and Table 2, respectively. More than half of the participants were between 21–24 years old (58% women; 54% men) and most identified as Christian (81% women; 83% men) and had Junior High School as the highest education level (55% women; 40% men). Most respondents were unmarried with a steady non-cohabitating partner (67% women; 73% men) and had never given birth (58%) or fathered a child (90%). The mean age of sexual debut was 17 for women and 16 for men, and both on average had been sexually active for four years. In total, 57% of men reported current modern contraceptive use with their partner whereas most women (54%) reported not using any contraceptive method. Women mostly had one to two lifetime sexual partners (71%), while men reported number of partners varied more widely with 36% reporting five or more lifetime sexual partners.

**Table 1.** Demographic and reproductive characteristics and knowledge of abortion law among young women, by abortion status (n=250) ^a^.

Characteristics	Overall (n=250)		Ever had abortion				p-value
			No (n=176, 70%)		Yes (n=74, 30%)		
	n	%	n	%	n	%	
Age, years	(Mean 21.1, IQR 4.0)		(Mean 20.9, IQR 4.0)		(Mean 21.6, IQR 4.0)		**0.006**
18–20	104	41.6%	83	47.2%	21	28.4%	
21–24	146	58.4%	93	52.8%	53	71.6%	
Highest education level							0.17
Junior high school	138	55.2%	93	52.8%	45	60.8%	
Senior high school	59	23.6%	48	27.3%	11	14.9%	
Tertiary	13	5.2%	7	4.0%	6	8.1%	
Vocational/technical	13	5.2%	10	5.7%	3	4.1%	
None	27	10.8%	18	10.2%	9	12.2%	
Religion							0.90
Christian	202	81.1%	143	81.3%	59	80.8%	
Muslim	46	18.5%	32	18.2%	14	19.2%	
None/traditional/spiritualist	1	0.4%	1	60.0%	0	0.0%	
Relationship status							
Married/cohabitating	71	28.4%	48	27.3%	23	31.1%	0.83
Steady partner, not living together	167	66.8%	119	67.6%	48	64.9%	
Single, divorced, widowed, or separated	12	4.8%	9	5.1%	3	4.1%	
Age of sexual debut	(Mean 17.2, IQR 3.0)		(Mean 17.4, IQR 3.0)		(Mean 16.5, IQR 3.0)		*0.008*
8–14	35	14.1%	17	9.7%	18	24.3%	
15–19	173	69.5%	126	72.0%	47	63.5%	
20–24	41	16.5%	32	18.3%	9	12.2%	
Number of lifetime sexual partners	(Mean 2.2, IQR 2.0)		(Mean 2.0, IQR 1.0)		(Mean 2.9, IQR 2.0)		*≤0.001*
1-2	177	71.1%	139	79.4%	38	51.4%	
3-4	56	22.5%	30	17.1%	26	35.1%	
5+	16	6.4%	6	3.4%	10	13.5%	
Ever given birth							0.73
Yes	104	41.6%	72	40.9%	32	43.2%	
No	146	58.4%	104	59.1%	42	56.8%	
Ever had a sexually transmitted infection test							0.36
Yes	110	44.2%	74	42.3%	36	48.6%	
No	139	55.8%	101	57.7%	38	51.4%	
Ever talked to anyone about family planning							0.75
Yes	134	54.3%	95	54.9%	39	52.7%	
No	113	45.8%	78	45.1%	35	47.3%	
Current family planning method							0.33
None	134	53.6%	95	54.0%	39	52.7%	
Modern method	97	38.8%	65	36.9%	32	43.2%	
Traditional method	19	7.6%	16	9.1%	3	4.1%	
Knowledge of abortion law							0.26
Legal in all conditions	14	5.7%	11	6.3%	3	4.1%	
Legal in some conditions	18	7.3%	9	5.1%	9	12.3%	
Illegal	101	40.7%	73	41.7%	28	38.4%	
Do not know	115	46.4%	82	46.9%	33	45.2%	

**Table 2.** Demographic and reproductive characteristics and knowledge of abortion law among young men, by partner abortion status (n=100) ^a^.

Characteristics	Overall (n=100)		Partner ever had abortion				p-value
			No (n=86, 86%)		Yes (n=14, 14%)		
	n	%	n	%	n	%	
Age, years	(Mean 20.7, IQR 3.0)		(Mean 20.6, IQR 3.0)		(Mean 21.7, IQR 3.0)		0.16
18–20	46	46.0%	42	48.8%	4	28.6%	
21–24	54	54.0%	44	51.2%	10	71.4%	
Highest education level							0.62
Junior high school	40	40.0%	34	39.5%	6	42.9%	
Senior high school	36	36.0%	29	33.7%	7	50.0%	
Tertiary	4	4.0%	4	4.70%	0	0.0%	
Vocational/technical	19	19.0%	18	20.9%	1	7.1%	
None	1	1.0%	1	1.2%	0	0.0%	
Religion							0.63
Christian	83	83.0%	72	83.7%	11	78.6%	
Muslim	14	14.0%	11	12.8%	3	21.4%	
None/traditional/spiritualist	3	3.0%	3	3.5%	0	0.0%	
Relationship status							
Married/cohabitating	8	8.0%	6	69.8%	2	14.3%	0.32
Steady partner, not living together	73	73.0%	62	72.1%	11	78.6%	
Single, divorced, widowed, or separated	19	19.0%	18	20.9%	1	7.1%	
Age of sexual debut	(Mean 16.3, IQR 3.0)		(Mean 16.4, IQR 3.0)		(Mean 15.8, IQR 3.0)		0.88
8–14	18	18.2%	15	17.7%	3	21.4%	
15–19	74	74.8%	64	75.3%	10	71.4%	
20–24	7	7.10	6	7.1%	1	7.1%	
Lifetime sexual partners	(Mean 5.0, IQR 4.0)		(Mean 4.7, IQR 4.0)		(Mean 7.2, IQR 5.0)		0.25
1-2	29	29.0%	27	31.4%	2	14.3%	
3-4	35	35.0%	31	36.1%	4	28.6%	
5+	36	36.0%	28	32.6%	8	57.1%	
Ever fathered a child							*0.004*
Yes	10	10.0%	5	5.8%	5	35.7%	
No	90	90.0%	81	94.2%	9	64.3%	
Ever had a sexually transmitted infection test							*0.03*
Yes	21	21.0%	15	17.4%	6	42.9%	
No	79	79.0%	71	82.6%	8	57.1%	
Ever talked to anyone about family planning		****					*0.04*
Yes	67	67.0%	61	70.9%	6	42.9%	
No	33	33.0%	25	29.1%	8	57.1%	
Current family planning method							0.32
None	26	26.0%	20	23.3%	6	42.9%	
Modern method	57	57.0%	51	59.3%	6	42.9%	
Traditional method	17	17.0%	15	17.4%	2	14.3%	
Knowledge of abortion law							0.77
Legal in all conditions	16	16.0%	13	15.1%	3	21.4%	
Legal in some conditions	20	20.0%	18	20.9%	2	14.3%	
Illegal	48	48.0%	42	48.8%	6	42.9%	
Do not know	16	16.0%	13	15.1%	3	21.4%	

^a^Numbers may not add up to totals due missing values. P-value < 0.05 considered significant and italicized in the table.

### Abortion knowledge and experience among women

While abortion in Ghana is legal in cases of rape, incest, risk to the life or health of the woman, or fetal abnormality when performed by a medical registered practitioner ^3^, 87% of women thought abortion was illegal or stated they did not know the law. Only 12% of women who had a prior abortion and 5% without a prior abortion thought it was legal in some cases. Of female respondents, 30% (n=74) reported ever having an abortion ( Table 1), with 21% (n=15) of them reporting more than one abortion over their lifetime (data not shown). 

In bivariate analyses ( Table 1), women who ever had an abortion were older (aged 21–24 versus 18–20, p=0.006), had an earlier sexual debut (aged 8–14 versus 15–19 versus 20–24, p=0.008), and reported more lifetime sexual partners (1–2 versus 3–4 versus 5+ partners, p≤0.001) compared to those who had never had an abortion. There were no significant univariate differences by education, religion, relationship status, parity, prior STI testing, past family planning discussion, family planning method, or knowledge of the abortion law.

When women were asked how they felt about being pregnant prior to their most recent or only abortion, most reported wanting to be pregnant later (69%), 15% reported they never wanted to be pregnant, and 15% reported wanting to be pregnant then ( Table 4). Women reported multiple reasons for seeking abortion, with the majority (74%) weighing the timing of their pregnancy as a driving factor. Timing included not being ready to be a mother (n=37), being too young to have a child (n=16), wanting to delay child bearing (n=7), and/or desiring child spacing (n=3). Other top reasons were for their career (36%), including that they wanted to stay in school (n=20) or continue working (n=6). Many also reported relationship reasons for ending their pregnancy (34%), which included the partner not wanting the child (n=21) or denying the pregnancy (n=2), not being in a serious relationship (n=1), or that the participant did not love the biological father (n=2). Others had concerns about resources (15%), including the lack of help (n=5) or money (n=6) for raising a child. In total, 15% said they sought their abortion due to their parents, either owing to fear of them (n=9) or because of their insistence (n=2) and 8% wanting to avoid shame. Only one participant mentioned concerns of health as a reason, which was due to risk of birth defects (1%).

Regarding support, most women spoke to someone about their abortion (89%), and 78% felt supported by their partner and/or 58% by their friends for their abortion. However, less than 30% reported that their family supported their abortion.

### Abortion methods

Women selected their initial or only abortion method for multiple reasons ( Table 4), with the most common reasons being the recommendation of family or friends (39%), their partner (22%), and/or a health provider (19%). Other reasons also included safety (16%), effectiveness (10%), privacy (3%), and/or because it was their only known method (14%).

Among the 74 women who reported prior abortion, the most common initial or only attempted method of abortion was medication abortion (n=45, 61%) ( Table 4). Specific methods initially sought included medication abortion (including misoprostol alone, referred to by the brand name “Cytotec” (n=34, 46%), and unspecified tablets (n=11, 15%)), surgical abortion (including D&C (n=14, 19%) and unspecified surgical abortion (n=5, 7%)), and unsafe methods (including consuming various drinks or liquids, ingesting herbal substances, inserting of herbs, objects, or substances into the vagina, and heavy massage (n=10, 14%)). Accounting for multiple methods used during women’s most recent abortion, 62% of women took tablets (n=46) and 32% had a D&C (n=24) at some point for their most recent abortion (data not shown). Demographic and sexual health characteristics by abortion method did not differ significantly except by religion (p=0.002) where reported (not reported by 1 respondent). Medication abortion was accessed by both Christian (n=30, 68%) and Muslim (n=14, 32%) women; however, D&C (n=19) and unsafe methods (n=10) were used as a first or only method by only Christian women (data not shown).

Generally, most women were very or somewhat satisfied with their initial or only chosen method ( Table 4). In total, 25% (n=18) reported any dissatisfaction in their initial method of surgical abortion (n=1), medication abortion (n=12), or unsafe methods (n=5). All those who reported being very dissatisfied with their initial method (n=10, 14%) used more than one method to end their pregnancy (data not shown).

One-fifth of respondents used more than one method (n=16, 22%). All women in this subset initially used medication abortion (n=11, 69) or unsafe methods (n=5, 31) with the other second method reported to be D&C (n=10, 63%), misoprostol (n=1, 6%), unspecified tablets (n=1, 6%), and unsafe methods (injection, drank herbal concoction, or heavy massage) (n=4, 25%). Nearly a quarter (n=11, 24%) of women who initially took tablets (misoprostol or unspecified) used more than one method, with most of them following up with D&C (n=7) (data not shown).

### Abortion providers

Women’s provider experiences for their most recent abortion are shown in Table 5. Among the 10 women whose initial method was unsafe, 50% saw no one, 10% saw a family member or friend, 20% saw a traditional practitioner, and 20% saw a doctor, nurse, or midwife. All women whose initial method was surgical abortion saw a doctor (n=17, 90%) or nurse/midwife (n=2, 9%) for their procedure at a medical facility. Conversely, only a few women (n=5, 11%) whose initial or only method was medication abortion visited a doctor, nurse, or midwife. Most of these women initially saw a pharmacist (n=18, 40%), while others consulted with friends/family (n=7, 16%) or no one (n=15, 33%). Overall, among all women with prior abortion, 55% (n=41) accessed medication abortion at some point outside of the formal medical system for their most recent abortion through a pharmacist (n=19), friends and family (n=7), or no one (n=15).

For the 73% of women (n=54) who reported visiting or consulting with someone (including non-professionals) for their abortion, most reported that recommendations from their family/friends (52%) factored into their decision regarding who they initially consulted with and where they went ( Table 5). Other common reasons were perceived quality of the provider (26%) and/or the recommendation of their partner (15%). Women who had surgical abortions were significantly more likely than those who had medication or other abortions to have considered quality when choosing their provider (p≤0.001).

When asked about satisfaction with where they went for their abortion, most women reported feeling satisfied, with satisfaction varying by method used (p=0.002) ( Table 5). All those who had surgical abortions were very or somewhat satisfied, while those who had medication abortions were more mixed. Only five respondents expressed any dissatisfaction with their initial location. Four had initially used medication abortion at home after visiting a pharmacist, while one saw a traditional healer for an herbal remedy. All five dissatisfied women used multiple methods to complete their recent abortion (data not shown).

A minority (39%) reported receiving any contraceptive information post-abortion; however, women who had surgical abortions were more likely to report talking about family planning with their providers compared to those who had medication abortions or used other methods (p=0.04). Overall, 62% of women who saw a health professional spoke to them before (n=6, 23%), after (n=8, 31%), or before and after (n=2, 8%) about post-abortion contraception. Of those who saw a pharmacist, 44% spoke about post-abortion contraception before (n=4) or after (n=4) their abortion. The majority (n=16, 80%) of women who saw no one received no post-abortion contraceptive counselling. Those who saw a traditional practitioner did not receive contraception counseling, while just one woman who saw a friend/relative reported talking with a health professional before her abortion about contraception (n=1, 13%) (data not shown).

Regarding costs, 54% reported their partners paid for the procedure, while 26% reported they paid for their own abortions. The cost for abortions varied widely. Surgical methods cost a median 150 GH¢ (n=15), significantly more than the median 30 GH¢ (n=26) for medication abortion and 55 GH¢ (n=6) for unsafe methods. Those who did not ever visit a provider were not asked about cost. 

### Abortion safety

Of the 74 women who reported ever having an abortion, 18% (n=13) reported using an unsafe method at some point during their recent abortion process, which included drinking substances, herbal remedies, inserting objects, taking an injection, or receiving a heavy massage. Among those who ever reported one of these unsafe methods, all of those who answered (n=12) did not know that abortion was legal. More women who used surgical or medication abortion were aware of the law. In fact, there was a significant difference in correct understanding of the law between those who reported using unsafe abortion methods versus surgical or medication abortion (p=0.046) (data not shown).

However, there were otherwise few significant differences between women who ever used an unsafe method versus surgical or medication abortion methods. Those who reported ever using an unsafe method were more likely to have more than two lifetime sexual partners (p=0.008). Also, women ever using unsafe abortion methods compared to medication abortion or surgical methods were more likely to report using more than one method to end their pregnancy (p=0.001), and to have reported seeing no one for their abortion (p=0.002). Women more often reported selecting an unsafe method due to assumed privacy (p=0.03) and/or effectiveness (p=0.02) of the method but were also more likely to be very dissatisfied with their method (p=0.003).

Despite unsafe methods being used, adverse events did not differ by method safety. Regarding additional required treatment, no one reported needing surgical intervention and two women reported receiving blood transfusions following a surgical abortion and a medication abortion respectively. Four women, two in each safety group, required antibiotics (data not shown). Four respondents reported health problems associated with the abortion after six months from their most recent abortion, while seven women had their abortion less than six months ago. Complaints included lack of period (n=1, 1%), abdominal pain (n=1, 1%), irregular period (n=3, 4%), more painful periods (n=1, 1%), and reported anemia (n=1, 1%) after six months. This included a woman who had a medication abortion, one who had a D&C, and two who used medication abortion followed by D&C or “injection” respectively (data not shown).

### Partner abortion characteristics

Among males, 64% thought abortion was illegal or stated they did not know the law ( Table 2). Only 14 (14%) reported that a partner had a prior abortion ( Table 2), with three (21%) of them reporting more than one prior abortion. Most respondents’ partners’ abortions were provided by a medical professional (n=9, 69%) and took place at a private clinic or hospital (n=8, 62%). Others noted that their partners saw a pharmacist (n=2, 15%) or a relative/friend (n=2, 15%) for their abortions (data not shown). Males reported that their partners had a medication (n=4, 29%) or surgical abortion (n=2, 14%); however, 57% (n=8) did not know or report what specific method was used (data not shown). Males who reported having a partner who had an abortion were more likely to have fathered a child (p=0.004) and ever been tested for an STI (p=0.03), and less likely to have ever spoken to anyone about family planning (p=0.04), compared to those who did not report a partner ever having an abortion ( Table 2). The majority (n=9, 64%) did not support their partner’s decision for abortion because they believed that it was dangerous for the woman (n=3), they wanted the child (n=1), they were not in a relationship with the pregnant person at the time of the abortion (n=2), or for no specified reason (n=4). None of the men said they believed their family would support the abortion, and only one mentioned talking to any family about it (data not shown).

## Discussion

Many respondents thought abortion was illegal or did not know the law, an unsurprising finding given that the 2017 Ghana Maternal Health Survey found only 11% of women of reproductive age believed abortion was legal ^16^. This, and past studies point to the lack of clarity in the law and need for further clarification and dissemination of the policy ^2^.

In total, 30% of women reported ever having an abortion in our study population. Medication abortion was the most used method, often accessed through a pharmacy or from friends and family. Overall, reported medication abortion was generally less expensive than surgical methods, and few women regardless of method reported any adverse health consequences. However, effectiveness and acceptability of medication abortion is less clear. Nearly a quarter of women who initially had a medication abortion sought a second method for completion. While this could be secondary to expected post-abortion bleeding. As a result, more needs to be done to assure that women know where to go for safe medication abortion and are receiving the appropriate doses and formulations of abortifacient medications at the correct time from trained, accessible providers. This speaks to a need for further training of pharmacists in the provision of medication abortion services. In addition, while overall women were satisfied with medication abortion, 27% reported dissatisfaction compared to 5% among women using surgical methods.

As previously noted, many participants, the majority of whom had medication abortions, reported not seeing anyone or seeing a family member or friend for their abortion. While home use of misoprostol is considered a possible safe option for women ^4^, it was unclear from these data where and from whom participants got their medications and exactly what formulation they ingested. Further, those who did not report seeing anyone were not given the same opportunities for post-abortion contraception counselling, a vital component of comprehensive abortion care. Fully assessing abortion safety, as previously noted, is challenging, and this study likely underestimates the actual prevalence of unsafe abortion as it does not consider the qualifications of the providers, the conditions of their facilities, or medication authenticity. Per our study definition however, nearly one-fifth of female respondents reported ever accessing an unsafe method for their abortion. These respondents were more likely to see no one and choose their method due to perceived privacy or effectiveness than those who used medication or surgical abortion, and all thought abortion was illegal, or did not know the law. Lack of understanding of the law and fear of prosecution could be factors pushing women to seek unsafe or informal means of abortion. Compounding this, a perceived lack of partner and familial support or fear of disclosing to them could further reduce the likelihood that an individual will seek out safe, medically appropriate services. While men were somewhat more knowledgeable of the law, they reported being unsupportive of their partners’ abortions. This speaks to a need to publicly correct misinformation around abortion and further destigmatize services.

Finally, surgical abortion, including most commonly D&C, was the second most utilized method, a finding consistent with methods identified in the 2017 Ghana Maternal Health Survey ^6^. We did not differentiate between suction and sharp curettage as the method of D&C; however, it should be noted that sharp curettage is not currently recommended by the World Health Organization ^4^, and efforts to replace it with vacuum aspiration should be further implemented ^17^

Our study is one of the few studies that assesses not only male perspectives on partner abortion, but also the reproductive health experiences and knowledge of young people in a non-medical setting. While abortion is often a sensitive, stigmatized topic, we were able to collect comprehensive information from both men and women in various locations in Accra. However, there are some limitations including the fact that we had a convenience sample and the data are self-reported and prone to both recall and selection bias. Also, much of the subgroup abortion analyses, particularly among males, were limited by small sample size. While the survey was comprehensive, the exact picture of where, how, and when women obtain medications for abortion and exactly what they consume remains unclear from our survey responses and merits further qualitative study. As can be seen from the Table 3, findings from our data are quite different from that of the Demographic and Health Survey (DHS) ^18^ – such as differences in terms of family planning method used and if the respondent had ever given birth/fathered a child. Hence it is not possible to generalize our findings.

While abortion is legal and safe services are available in Ghana, more needs to be done to improve access through correcting misinformation and informing all people not only of the law, but also where one can privately and safely receive appropriate care.

**Table 3.** Comparison of data with Demographic Health Survey (2014).

Characteristics	Female		Male	
	Our study	DHS (2014)	Our study	DHS (2014)
**Highest education level**				
Junior high school / Some secondary (DHS) 15-24	55.2%	53.8%	40.0%	59%
Senior high school / Completed secondary (DHS)	23.6%	15.7%	36.0%	17.3%
Tertiary / More than secondary (DHS)	5.2%	4.0%	4.0%	4.1%
Vocational/technical	5.2%	NA	19.0%	NA
None	10.8%	8.1%	1.0%	3.2%
**Relationship status**				
Married/cohabitating	28.4%	21.9%	8.0%	4.5%
Steady partner, not living together	66.8%	NA	73.0%	NA
Single, divorced, widowed, or separated /Never married, divorced or separated, widowed (DHS)	4.8%	75.4%	19.0%	95.5%
**Age of sexual debut**				
8–14	14.1%	NA	18.2%	NA
15–19	69.5%	32.3%	74.8%	20.5%
20–24	16.5%	35.9%	7.10%	65.0%
**Ever given birth / Ever fathered a child**				
Yes	41.6%	30.4%	10.0%	NA
No	58.4%	70.7%	90.0%	NA
**Current family planning method**				
None	53.6%	81.4%	26.0%	NA
Modern method	38.8%	14.0%	57.0%	NA
Traditional method	7.6%	4.9%	17.0%	NA

NA: No comparable question/category included in the survey

**Table 4.** Abortion experiences among young women at most recent abortion, by initial or only abortion method (n=74) ^a^.

Characteristics	Overall (n=74)		Initial or only recent abortion method ^b^						p-value
			Medication abortion (n=45, 61%)		Surgical abortion (n=19, 26%)		Unsafe safe method (n=10, 14%)		
	n	%	n	%	n	%	n	%	
1st trimester abortion	62	86.1%	38	86.4%	14	77.8%	10	100.0%	0.33
More than one method	16	21.6%	11	24.4%	0	0.0%	5	50.0%	*0.003*
Wanted to be pregnant:									0.152
Then	11	15.3%	4	88.9%	6	35.3%	1	10.0%	
Later	50	69.4%	34	75.6%	9	52.9%	7	70.0%	
Not at all	11	15.3%	7	15.6%	2	11.8%	2	20.0%	
Reason for abortion: ^c^									
Timing	54	74.0%	35	77.8%	11	61.1%	8	80.0%	0.34
Career	26	35.6%	15	33.3%	7	38.9%	4	40.0%	0.83
Relationship reasons	25	33.8%	14	31.1%	6	31.6%	5	50.0%	0.50
Lack of resources	11	15.1%	7	15.6%	2	11.1%	2	20.0%	0.80
Parents	11	15.1%	6	13.3%	3	16.7%	2	20.0%	0.72
To avoid shame	6	8.2%	4	8.9%	1	5.6%	1	10.0%	1.00
Health concerns of fetus	1	1.4%	0	0.0%	1	5.3%	0	0.0%	0.39
Reason for method: ^c^									
Recommended by family/friends	29	39.2%	18	40.0%	6	31.6%	5	50.0%	0.58
Recommended by partner	16	21.6%	12	26.7%	3	15.8%	1	10.0%	0.51
Recommended by healthcare provider	14	18.9%	12	26.7%	2	10.5%	0	0.0%	0.09
Safety	12	16.2%	2	4.4%	10	52.6%	0	0.0%	*≤0.001*
Only known method	10	13.5%	5	11.1%	4	21.1%	1	10.0%	0.62
Effectiveness	7	9.5%	3	6.7%	0	0.0%	4	40.0%	*0.004*
Privacy	2	2.7%	0	0.0%	0	0.0%	2	2.7%	*0.02*
Method satisfaction:									0.08
Very satisfied	40	54.8%	21	47.7%	14	73.7%	5	50.0%	
Somewhat satisfied	13	17.8%	10	22.7%	3	15.8%	0	0.0%	
Somewhat dissatisfied	8	11.0%	6	13.6%	1	5.3%	1	10.0%	
Very dissatisfied	10	13.7%	6	13.6%	0	0.0%	4	40.0%	
Doesn’t know	2	2.7%	1	2.3%	1	5.30%	0	0.0%	
Spoke to anyone about their abortion	65	89.0%	40	90.9%	18	95.0%	7	70.0%	0.12
Abortion Support: ^c^									
Friend supported	42	57.5%	28	63.6%	10	52.6%	4	40.0%	0.39
Family supported	20	27.4%	10	22.7%	6	31.6%	4	40.0%	0.49
Partner supported	57	78.1%	34	77.3%	15	79.0%	8	80.0%	1.00
Provider supported	42	58.3%	20	45.5%	17	94.4%	5	50.0%	*≤0.001*

^a^Numbers may not add up to total due missing values. P-value < 0.05 considered significant and bolded in the table. ^b^Medication abortion included misoprostol or unspecified tablets; surgical abortion included surgical methods and dilation and curettage; unsafe method included drinks or other liquids, ingesting herbal substances, injection, insertion of herbs, objects, or substances into the vagina, and heavy massage. ^c^More than one response allowed.

**Table 5.** Provider experiences among young women, by initial or only abortion method at most recent abortion (n=74) ^a^.

Characteristics	Overall (n=74)		Initial or only recent abortion method ^b^						p-value
			Medication abortion n=45 (61%)		Surgical abortion n=19 (26%)		Unsafe method n=10 (14%)		
	n	%	n	%	n	%	n	%	
Abortion provider									*≤0.001*
Doctor, nurse, or midwife	26	35.1%	5	11.1%	19	100.0%	2	20.0%	
Pharmacist	18	24.3%	18	40.0%	0	0.0%	0	0.0%	
Traditional provider	2	2.7%	0	0.0%	0	0.0%	2	20.0%	
Relative/friend	8	10.8%	7	15.6%	0	0.0%	1	10.0%	
No one	20	27.0%	15	33.3%	0	0.0%	5	50.0%	
Location satisfaction									*0.002*
Very satisfied	36	50.0%	16	37.2%	16	84.2%	4	40.0%	
Somewhat satisfied	11	15.3%	8	18.6%	3	15.8%	0	0.0%	
Somewhat dissatisfied	3	4.2%	2	4.7%	0	0.0%	1	10.0%	
Very dissatisfied	2	2.8%	2	4.7%	0	0.0%	0	0.0%	
Does not know	1	1.4%	0	0.0%	0	0.0%	1	10.0%	
Did not go anywhere	19	26.4%	15	34.9%	0	0.0%	4	40.0%	
Discussed family planning at abortion with health worker	29	39.2%	15	33.3%	12	63.2%	2	20.0%	*0.04*
*Among those who saw someone (n=54)*									
Reason for provider choice ^c^									
Family/friend recommended	28	51.9%	17	56.7%	9	47.4%	2	40.0%	0.72
Quality	14	25.9%	1	3.3%	12	63.2%	1	20.0%	*≤0.001*
Partner recommended	8	14.8%	5	16.7%	1	5.3%	2	40.0%	0.13
Cost	5	9.3%	5	16.7%	0	0.0%	0	0.0%	0.20
Privacy	7	13.0%	5	16.7%	1	5.3%	1	20.0%	0.38
Distance	4	7.4%	2	6.7%	2	10.5%	0	0.0%	0.76
Abortion location									*≤0.001*
Home	20	37.0%	18	60.0%	0	0.0%	2	40.0%	
Public sector	6	11.1%	0	0.0%	4	21.1%	2	40.0%	
Private sector	21	38.9%	5	16.7%	15	79.0%	1	20.0%	
Pharmacy	7	13.0%	7	23.3%	0	0.0%	0	0.0%	
Who paid for the abortion									0.49
Self	14	25.9%	9	30.0%	5	26.3%	0	0.0%	
Partner	29	53.7%	15	50.0%	10	52.6%	4	80.0%	
Mother	2	3.7%	1	3.3%	1	5.3%	0	0.0%	
Other family	5	9.3%	3	10.0%	2	10.5%	0	0.0%	
Friend	2	3.7%	2	6.7%	0	0.0%	0	0.0%	
Self and other family	1	1.9%	0	0.0%	0	0.0%	1	20.0%	
Partner and mother	1	1.9%	0	0.0%	1	5.3%	0	0.0%	
Total cost of abortion (Gh¢) (n=43)									*≤0.001*
<50	18	41.9%	15	60.0%	1	6.7%	2	66.7%	
50–149.99	15	34.9%	9	36.0%	5	33.3%	1	33.3%	
150+	10	23.3%	1	4.0%	9	60.0%	0	0.0%	

^a^Numbers may not add up to total due missing values. P-value < 0.05 considered significant and bolded in the table. ^b^Medication abortion included misoprostol or unspecified tables; surgical abortion included surgical methods and dilation and curettage; unsafe method included drinks or other liquids, ingesting herbal substances, injection, insertion of herbs, objects, or substances into the vagina, and heavy massage. ^c^More than one response allowed.

## Data availability

### Underlying data

Harvard Dataverse: Replication Data for: Abortion knowledge and experiences among young women and men in Accra, Ghana. https://doi.org/10.7910/DVN/ZBZT1N ^19^.

This project contains the following underlying data:

- Ghana Survey_Abdata.tab (underlying data)
- Ghana Survey_Ab data dictionary.do (data dictionary)

### Extended data

Harvard Dataverse: Replication Data for: Abortion knowledge and experiences among young women and men in Accra, Ghana. https://doi.org/10.7910/DVN/ZBZT1N ^19^.

This project contains the following extended data:

- Survey_female (survey given to female participants)
- Survey_male (survey given to male participants)

Data are available under the terms of the Creative Commons Zero "No rights reserved" data waiver (CC0 1.0 Public domain dedication).
