# Supplementary material for: Abortion knowledge and experiences among young women and men in Accra, Ghana
Source: Gates Open Res. 2020 Sep 7;3:1478. Originally published 2019 May 30. [Version 2] doi: 10.12688/gatesopenres.12961.2 (PMC7498762; doi:10.12688/gatesopenres.12961.2)
Supplement: Peer review history

# Peer review: Reviewer response for version 2

**DOI:** 10.21956/gatesopenres.14287.r29501

**Published:** 2020-09-17

**Authors:** Suzanne O. Bell

**Affiliations:** 1 Department of Population, Family and Reproductive Health, Johns Hopkins Bloomberg School of Public Health (JHSPH), Baltimore, MD, USA

The authors have done a good job responding to reviewer comments and have improved the paper. However, one remaining area where I am still unclear is the sampling. Authors added no additional information, that I can see, other than to indicate the cross-sectional surveys were administered "randomly". Random has a very specific connotation in survey research and from the available methods description, it does not appear the survey participants were in fact selected randomly. If interviewers simply approached people at random (which is different) and did not track days/times and response rates, it would be more accurate to say the survey was administered to a convenience sample. Authors should use this terminology in the methods section, as well as the limitations section and the abstract to make it explicit to readers. Otherwise I approve this manuscript for indexing.

Is the work clearly and accurately presented and does it cite the current literature?

Yes

If applicable, is the statistical analysis and its interpretation appropriate?

Partly

Are all the source data underlying the results available to ensure full reproducibility?

Yes

Is the study design appropriate and is the work technically sound?

Partly

Are the conclusions drawn adequately supported by the results?

Partly

Are sufficient details of methods and analysis provided to allow replication by others?

No

Reviewer Expertise:

NA

I confirm that I have read this submission and believe that I have an appropriate level of expertise to confirm that it is of an acceptable scientific standard.

# Peer review: Reviewer response for version 2

**DOI:** 10.21956/gatesopenres.14287.r29502

**Published:** 2020-09-07

**Authors:** Allan G. Hill

**Affiliations:** 1 Department of Social Statistics and Demography, Faculty of Social and Human Sciences, University of Southampton, Southampton, UK

The authors have responded accurately and efficiently to all suggestions and comments. I hope to see the work in print soon.

Is the work clearly and accurately presented and does it cite the current literature?

Yes

If applicable, is the statistical analysis and its interpretation appropriate?

Yes

Are all the source data underlying the results available to ensure full reproducibility?

Yes

Is the study design appropriate and is the work technically sound?

Yes

Are the conclusions drawn adequately supported by the results?

Yes

Are sufficient details of methods and analysis provided to allow replication by others?

Yes

Reviewer Expertise:

Demography and international health; current work on the health of women in West Africa; fertility and mortality transitions in West Africa

I confirm that I have read this submission and believe that I have an appropriate level of expertise to confirm that it is of an acceptable scientific standard.

# Peer review: Reviewer response for version 1

**DOI:** 10.21956/gatesopenres.14067.r28471

**Published:** 2020-02-03

**Authors:** Suzanne O. Bell

**Affiliations:** 1 Department of Population, Family and Reproductive Health, Johns Hopkins Bloomberg School of Public Health (JHSPH), Baltimore, MD, USA

**Overall comments**

 The paper provides interesting insights into abortion among youth in Accra, Ghana, however, the generalizability of these findings is limited given the sampling approach. Authors should further describe the sampling/recruitment method and make more explicit that findings are likely subject to bias and should be interpreted with caution. If possible, authors should compare sample characteristics to that of a larger, representative population of similarly aged people in Accra (e.g. Ghana Maternal Health Survey) to assess selection bias. Nonetheless, the conclusion that knowledge of the law and widespread use of unsafe abortion methods is apparent, regardless of extent of biases. I provide specific major and minor comments below.

**Specific comments**

 Major comments

*Methods*

- Study design and sample: Provide more detail on how interviewers approached/recruited eligible participants from recruitment sites. Did they approach everyone? Did they randomly select every Xth person who entered? Did they vary the days/times they recruited? Or was it just a convenience sample? Also, what was the response rate among men and women who interviewers approached to participate in the survey?
- Data analysis: Since not actually using WHO “least safe” definition, would recommend just referring to category as unsafe or non-recommended methods; not factoring in setting/provider, as authors indicate, which is key aspect of WHO definition.
- Did authors examine differences in characteristics in their survey to those in other survey? For example, Ghana Maternal Health Survey? Would recommend doing so to provide assessment of how different this sample is from a comparable population that was sampled in a representative manner.
- Would be helpful to also provide sociodemographic characteristics of women in comparison of characteristics by initial or only recent abortion method; from a public health and health equities perspective, important to know if differences in safety of abortions are significantly related to women’s sociodemographic characteristics, not just characteristics specific to the abortions. 

*Discussion*

- With regards to need for subsequent method following medication abortion, this could simply be normal bleeding. Women may be concerned and present for care even though the abortion is progressing normally without complications. Need to mention this in explanation/discussion of the nearly one-quarter of women using medication abortion who received subsequent care prior to completion.
- I would not necessarily consider medication abortion (if using MA drugs, not other medications) “less safe”. If using less safe terminology in relation to WHO definition, would specify as such and define “less safe” criteria. Again, since not incorporating source/provider conditions and training, would consider using different terminology so not applying same terms to different construct.
- Authors include no discussion of study limitations with regard to generalizability/representativeness. Given the recruitment method, we cannot generalize findings to a broader population in the Accra area. The 30% of women who had ever had an abortion is likely biased given the sampling design and we know direct questions result in significant underreporting (which authors briefly mention in second to last paragraph of discussion). The measures of abortion method/safety are likely also biased if there is differential underreporting of abortion by method.

Minor comments

*Abstract*

- “In total, 30% of women and 14% of men ever had an abortion and partner who had an abortion, respectively”: To improve clarity, would recommend rephrasing: “In total, 30% of women reported ever having an abortion and 14% reported ever having a partner who had an abortion”. 

*Background*

- Page 3: Missing end quote following unsafe abortion definition: In fact, unsafe abortion, defined as ‘a procedure for terminating an unintended pregnancy carried out either by persons lacking the necessary skills or in an environment that does not conform to minimum medical standards, or both4, is the second single leading cause of maternal death in Ghana.
- Rephrase second part of “As part of this effort, more was done to disseminate the law” to “…disseminate information about the abortion law…”
- Rephrase from “an already Food and Drug Board-approved oxytocic drug” to “an oxytocic drug already approved by the Food and Drug Board”. 

*Methods*

- Study design and sample: Unclear what “two paired teams of trained fieldworkers” means.
- Study design and sample: How did interviewers ask about abortion? Did questions use “induced abortion” terminology or equivalent translation? Specify.
- Data analysis: change “reported prior abortion” to “reported a prior abortion” in the following sentence: “For this study, our primary outcomes of interest were the percentage of women who reported prior abortion…”
- Data analysis: for consistency with how authors describe response categories for medication abortion and surgical, should describe what is included in “least safe” based on response options, not general definition from WHO.
- Data analysis: provide clearer explanation of analysis. Do not specify that chi-squared tests are in relation to bivariate analyses examining differences by whether reported prior abortion; that analyses are stratified by sex; that conducted bivariate analyses by first/only method of abortion.

*Results*

- Table 1: p-value assessing difference in average age is bolded instead of italicized.
- Table 2: Typo in age of sexual debut category “20–s24”.
- Abortion knowledge and experience among young women: Second paragraph should begin “among bivariate analysis…”
- Regarding support, most women spoke to someone about their abortion (89%), and 78% felt supported by their partner and/or 58% by their friends for their abortion.” Remove “or” from “and/or”.
- “However, most (73%) also reported that their family did not know about or did not support their abortion.” Can you distinguish between family not knowing and not supporting? Seems like an important distinction.
- Would separate “unspecified tablets” from “medication abortion” in the results; the phrase “medication abortion” connotes miso with or without mife. Including “unspecified tablets” will artificially inflate the MA estimate. Alternatively, could refer to category as abortion using tablets, or something to that effect.
- “Accounting for multiple methods  used during women’s most recent abortion, 62% of women took tablets (n=46) and 32% had a D&C (n=24)”: how was this calculated? If a woman reported using tablets at any point, was she categorized as using tablets? Same with D&C?
- Can remove repeated reference to “among women who had abortions” or the like once referring to specifics of abortions; clear that could not include responses from women who had not had abortions.
- “The majority (n=16, 80%) of women who saw no one received no post-abortion contraceptive counselling”: How can someone receive postabortion contraceptive counseling if they saw no one? Who is the counseling from? Clarify.
- “In fact, there was a significant difference  in correct understanding of the law between those who reported using least safe abortion methods versus surgical or medication abortion (p=0.046) (data not shown).”: specify that those who used surgical or MA were more likely to have correct knowledge (I assume this is the finding based on the fact that 0 women used least safe methods reported correct knowledge).

 
*Discussion*

- Specify percent who did not know about abortion law when comparing to GMHS results for ease of comparison.

Is the work clearly and accurately presented and does it cite the current literature?

Yes

If applicable, is the statistical analysis and its interpretation appropriate?

Partly

Are all the source data underlying the results available to ensure full reproducibility?

Yes

Is the study design appropriate and is the work technically sound?

Partly

Are the conclusions drawn adequately supported by the results?

Partly

Are sufficient details of methods and analysis provided to allow replication by others?

No

Reviewer Expertise:

Abortion measurement, survey research

I confirm that I have read this submission and believe that I have an appropriate level of expertise to confirm that it is of an acceptable scientific standard, however I have significant reservations, as outlined above.

# Response

**Published:** 2020-04-28

**Authors:** Sruthi Chandrasekaran

Thank you for your comments and detailed feedback. As per your feedback, we have incorporated the following overall changes:

1. Included a table comparing characteristics of men and women in our sample with comparable age groups in the DHS survey for Ghana to clarify the extent to which findings can be generalized. 

2. Provided further description on sampling and recruitment methods.

3. Changed to using "unsafe method" throughout, as per your recommendation.

4. Clarified the text based on your major and minor feedback where possible. 

5. The questions used are referenced in the survey instrument attached to the manuscript. 

6. Yes, if tablets were used at any point, the respondent was categorizes as using tablets, similarly with D&C. It could potentially be more than 100%.

# Peer review: Reviewer response for version 1

**DOI:** 10.21956/gatesopenres.14067.r27293

**Published:** 2019-06-11

**Authors:** Allan G. Hill

**Affiliations:** 1 Department of Social Statistics and Demography, Faculty of Social and Human Sciences, University of Southampton, Southampton, UK

This is a report on a small study on induced abortion amongst 250 women and 100 men aged 18-24 in Accra, Ghana. The topic is important since we believe abortion to be more widely used than reported in many low fertility urban populations in Africa but the methods and motivations are less well known. Ghana is a good case study since its laws on induced abortion are relatively liberal. The study adds some new information about abortion knowledge, provides descriptive information on methods used, providers and safety.

- In such small studies, the question is often how representative are the findings. A useful addition therefore might be to compare the characteristics of the men and women in the sample (Tables 1 and 2) with people of the age groups in the DHS survey data for Accra.
- Some small changes - maybe use “unsafe method” for “least safe method” in the section on abortion safety?
- It would be useful to add a note about the low fertility in Accra - authors' other publications - and the curious combination of low fertility and relatively low use of modern contraception.
- Other work by Oliveras *et al.* might be cited to show that abortion is also used by older higher parity women in Accra.

Otherwise, this is a well-written and clear report on the survey, containing some new information on the use of induced abortion whilst pointing to some possible interventions around improving knowledge and access.

Is the work clearly and accurately presented and does it cite the current literature?

Yes

If applicable, is the statistical analysis and its interpretation appropriate?

Yes

Are all the source data underlying the results available to ensure full reproducibility?

Yes

Is the study design appropriate and is the work technically sound?

Yes

Are the conclusions drawn adequately supported by the results?

Yes

Are sufficient details of methods and analysis provided to allow replication by others?

Yes

Reviewer Expertise:

Demography and international health; current work on the health of women in West Africa; fertility and mortality transitions in West Africa.

I confirm that I have read this submission and believe that I have an appropriate level of expertise to confirm that it is of an acceptable scientific standard.

# Response

**Published:** 2020-04-28

**Authors:** Sruthi Chandrasekaran

Thank you for your comments and feedback. We have incorporated a table comparing characteristics of men and women in our sample with comparable age groups in the DHS survey for Ghana. We also changed to using "unsafe method" throughout, as per your recommendation.
